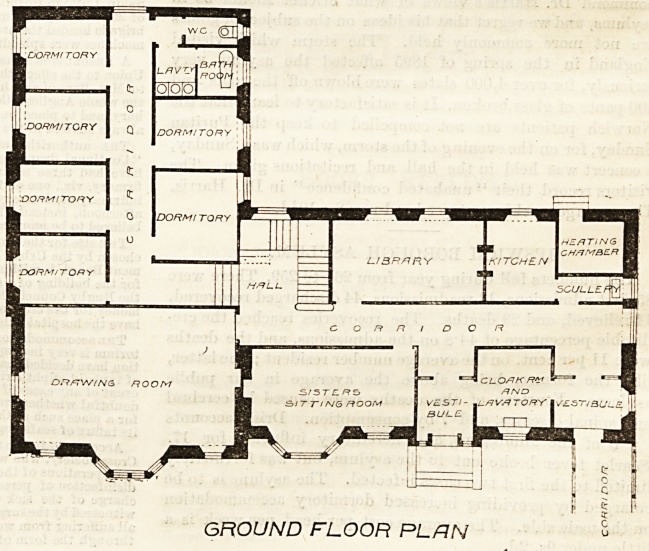# Hospital Construction

**Published:** 1896-10-24

**Authors:** 


### The Institutional Workshop.

### HOSPITAL CONSTRUCTION.

### NURSES' HOME, ROYAL INFIRMARY, DUNDEE.

This building, standing within the grounds of the infirmary, and connected with it by a glazed corridor, has been designed by Mr. Alexander Johnston, of Dundee. It is L-shaped on plan.

FIRST AND SECOND FLOORS

The second floor is nearly identical in arrangement with the first floor (a plan of which we publish), each containing fifteen dormitory rooms for nurses, with bath-rooms, lavatories, and w.c.'s. Corridors six feet in width, and lighted at the ends, give access to the various rooms, one staircase serving the whole of the building. On the second floor a sick-room is provided above the head-sister's sitting and bed room shown on the first floor.

On the ground floor there is additional bedroom accommodation for six nurses, with bath-room and w.c., the remainder of this floor being devoted to a drawing-room, 31 ft. by 20 ft., a library, a sisters' sitting-room, a kitchen and small scullery, a general entrance to the home, and a separate entrance from the infirmary, with a cloak-room well arranged between the two entrances. The corridors on this floor do not seem so well managed in respect of lighting as on the floors above.

The general warming of the building is by steam from a heating chamber on the ground floor, but fireplaces are provided in addition in the sitting-rooms and in the library. The ventilation is described by the architect as effected "by gratings in the foot-base in corridors and shafts up partitions to exhaust-ventilators in roofs.'

The general arrangement of the building appears simple and practical, but direct air and light should have been (and in several cases might have been) secured to the lobbies between the sanitary appliances and the corridors, while there seems no valid reason for having two sets of these arrangements on each floor, which has necessitated placing some of them in the middle of the building.

It is assumed that the screen between the bath-rooms and the lavatories is a low one, and does not extend to the ceiling of the apartment, as the plans appear to indicate, and that the lavatory basins do not, therefore, stand in an unventilated box. The dormitory rooms are well proportioned and sufficiently, without being extravagantly, large; but the adoption of a central corridor system and an L-shaped plan gives some of the rooms the benefit of the sun and deprives others of it altogether. Other points in these plans open to some criticism are that the sick-room can hardly be (as it ought to be for every reason in such an institution) carefully isolated and provided with separate sanitary appliances apart from the rest of the house, and that the special arrangements necessary for accommodating the night staff apart from the day nurses are not clearly worked out.

GROUND FLOOR PLAN

## Figures and Tables

**Figure f1:**
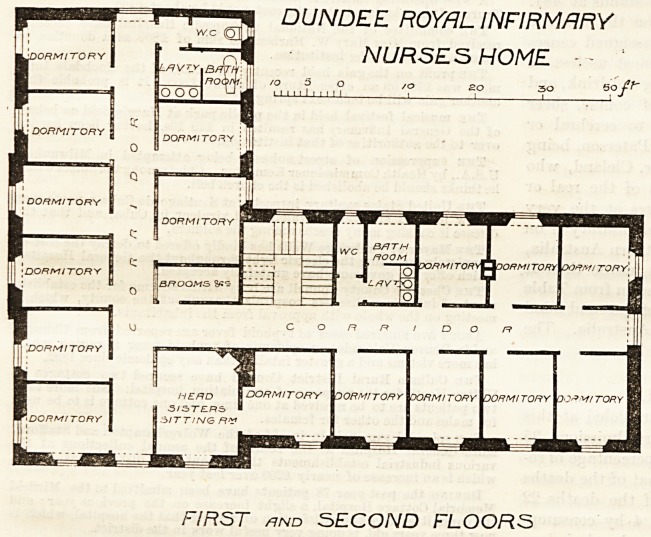


**Figure f2:**